# Association between use of different long-term care services and risks of mental disorder and mortality as well as medical utilization

**DOI:** 10.3389/fpsyt.2023.1073030

**Published:** 2023-10-02

**Authors:** Pei-Ying Tseng, Chia-Ling Wu, Jen-De Chen, Kai-Jie Ma, Chi-Yu Yao, Jong-Yi Wang

**Affiliations:** ^1^Department of Public Health, China Medical University, Taichung, Taiwan; ^2^Department of Medical, Lee’s General Hospital, Miaoli, Taiwan; ^3^Hospital Accreditation Department, Joint Commission of Taiwan, Taipei, Taiwan; ^4^Department of Sports, National Changhua University of Education, Changhua, Taiwan; ^5^Department of Psychiatry, An-Nan Hospital, Tainan, Taiwan; ^6^Department of Health Services Administration, China Medical University, Taichung, Taiwan

**Keywords:** long-term care, mental disorder, mortality, medical utilization, health maintenance

## Abstract

**Objective:**

This study sought to investigate mental disorder and mortality risks and medical utilization among various long-term care (LTC) services and examine the associated factors.

**Methods:**

This retrospective cohort study used data from the National Health Insurance Research Database of the entire population of Taiwan recorded between 2006 and 2017. A total of 41,407 patients using LTC (study group) were identified and propensity score–matched with 41,407 LTC nonusers (control group) at a ratio of 1:1 according to sex, age, salary-based premium, comorbidity index score, and urbanization level. Patients were divided into four groups according to LTC service type. The age distribution was as follows: 50–60 years (10.47%), 61–70 years (14.48%), 71–80 years (35.59%), and 81 years and older (39.45%). The mean age was 70.18 years and 53.57% of female participants were included. The major statistical methods were the Cox proportional hazards model and the general linear model (GLM).

**Results:**

Users of both institutional and inhome LTC services had the highest risk of mental disorder [adjusted hazard ratio (aHR) = 3.2]. The mean mortality rate in LTC nonusers was 46.2%, whereas that in LTC users was 90.4%, with the highest found among the users of both institutional and inhome LTC (90.6%). The institutional LTC users had the shortest survival time (4.1 years). According to the adjusted Cox model analysis, the odds of mortality was significantly higher among institutional LTC users than among inhome LTC users (aHR = 1.02). After the adjustment of covariates, adjusted GLM model results revealed that the annual medical expenditure *per capita* of LTC nonusers was NT$46,551, which was 1.6 times higher that of LTC users.

**Conclusion:**

Users of both institutional and inhome LTC services have higher risk of mental disorder, shorter survival time, and lower medical utilization.

## 1. Introduction

The issue of population aging has garnered significant attention worldwide. Remarkable advances in medical technology have led to reduced mortality rates, increased life expectancy, and a decline in birth rates, resulting in a phenomenon termed demographic aging. According to the World Health Organization, an “aging society” is characterized by individuals aged over 65 years comprising more than 7% of the total population. Taiwan officially entered this stage in 1993, with individuals aged over 65 years accounting for 14.1% of the national population by the end of March 2018. Projections indicate that this percentage will rise to 20% by 2026, making Taiwan a “super-aged society” (1). Consequently, the prevalence of chronic diseases is expected to increase as the older population rapidly expands, leading to an on-going rise in the population with disabilities.

The development of mental illness is common among older adults living in long-term care (LTC) facilities, with prevalence rates ranging from 0.5 to 64.7% (refer to the literature review below). Several studies have highlighted that the rate of comorbid mental illness is higher for older adults living in LTC facilities compared to older adults living in communities (2, 3). The chronic nature of mental illness further exacerbates the challenges faced by affected individuals. Considering the significant increase in elderly population, the utilization of LTC facilities has become increasingly critical for addressing the complex mental health needs of this vulnerable population. Elderly as well as young-disabled individuals who meet the qualifications are eligible for both inhome and institutional LTC services.

The primary objective of this study is to analyze the factors associated with mental illness, mortality risk, and healthcare costs among patients receiving LTC services. By understanding the importance of utilizing different types of LTC services in preventing mental illness, effective interventions can be implemented to reduce the risk of developing such conditions. Additionally, appropriate resource allocation to different types of LTC services can enhance their accessibility, localization, and overall service quality. To achieve this, nationwide representative data were utilized in this study to examine the effects of employing various types of LTC services on the risk of mental illness, mortality rates, and healthcare costs.

The findings of this study can significantly contribute to this area of research. This study generates valuable insights into the complex interplay between different types of LTC services, mental illness risk, mortality rates, and healthcare expenditure by comprehensively analyzing nationwide representative data. Such insights can inform evidence-based interventions and resource allocation strategies, ultimately leading to improved mental health outcomes and overall well-being among patients receiving LTC services.

In summary, this study addresses the pressing issue of population aging and its implications on the mental health outcomes among individuals in LTC facilities. By utilizing rigorous methodologies and nationwide representative data, the work advances our understanding of the factors influencing mental illness, mortality risk, and healthcare costs in this population. Moreover, the findings of this study have the potential to inform interventions, enhance resource allocation, and contribute to existing information in this area, ultimately improving the quality of LTC services and the overall well-being of individuals in an aging society.

### 1.1. Literature review

LTC facilities play a critical role in providing comprehensive care to individuals with functional decline and dependency. Understanding the factors that influence the choice of LTC facility service types is essential for optimizing care delivery and improving patient outcomes. This literature review aims to analyze and differentiate existing evidence from international and US studies with a focus on mortality rates, healthcare costs, and the prevalence of mental disorders among patients in institutional and community-based care settings. We have also explored the influence of sex, age, income, comorbidity severity, cancer incidence, and urbanization levels on LTC service preferences. By examining these factors, this review aims to highlight the complexities of LTC decisions and identify potential avenues for future research and policy development.

### 1.2. Mortality rates and healthcare costs

Previous studies have reported that the average survival time for patients admitted to LTC facilities ranges from 2.2 to 5.7 years (4, 5). Notably, the mortality rate of individuals utilizing institutional care services is significantly higher (71.6%) that that of individuals opting for community-based care services (58.8%) (6). This disparity in mortality rates underscores the importance of carefully considering the choice of care setting for LTC recipients.

In terms of healthcare costs, the financial burden associated with institutional care services is considerably higher than that of inhome care services. Patients utilizing institutional care services spend approximately 3,280 US dollars (USD) per month, whereas those availing inhome care services incur substantially lower costs of around 461 USD per month (7). These findings have significant implications on healthcare planning and resource allocation, emphasizing the need to strike a balance between cost-effectiveness and the quality of care being provided.

### 1.3. Prevalence of mental disorders

Mental health is a critical aspect of LTC, and the prevalence of mental disorders varies across different care settings. A former study reported that among common mental disorders in individuals using inhome care services, 28% will develop depression and 18.9% will experience anxiety disorders (8). In contrast, the prevalence rates for depression, anxiety disorder, schizophrenia, severe depression, and bipolar disorder among individuals utilizing community-based care services are 7.8, 4.8, 1.4, 1.2, and 0.6%, respectively (9). For individuals living in institutional care settings, the rates vary 6–71% for dementia, 24–37% for depression, 4–35% for severe depression, 8.3% for anxiety disorder, 4% for schizophrenia, and 1.9% for bipolar disorder (2, 9–12).

International studies have further highlighted that the lifetime prevalence rate of severe depression is approximately 20.4% in older females and approximately 9.6% in older males, indicating higher susceptibility among females (13). The risk of developing mental illness increases with age (14) and is more prevalent among individuals with low incomes receiving inhome and community-based care services (15). Additionally, the severity of comorbidities, as measured by the Charlson comorbidity index (CCI), negatively correlates with the risk of developing mental illness (8, 16). However, it is worth noting that as the CCI score increases, the incidence rate of severe depression rises, with the highest rates observed among those scoring more than 3 points on the CCI (17).

### 1.4. Factors influencing LTC service choices

The decision to utilize LTC services is influenced by various factors including sex, age, income, comorbidity severity, cancer incidence, and urbanization levels. With increasing age, individuals experience functional decline and increased dependency, leading to a greater demand for institutional care services (4, 18). Individuals in need of care or their family members may consider the severity of their physical condition, economic status, and personal preferences when choosing between different care settings.

### 1.5. Inconsistencies in previous findings

While this literature review provides valuable insights regarding the factors influencing LTC service choices and their associated outcomes, the inconsistencies across studies are noteworthy. Factors such as sex, age, income, comorbidity severity, cancer incidence, and urbanization levels have been identified as influential determinants, yet the findings regarding their specific impact on care preferences vary. Such inconsistencies highlight the necessity for further investigation and clarification to establish more robust associations between these factors and LTC decisions.

### 1.6. Summary

This comprehensive literature review has provided an overview of key findings related to mortality rates, healthcare costs, and mental disorder rates among individuals receiving LTC in different settings. The evidence underscores the higher mortality rates and healthcare costs associated with institutional care services, as well as the higher prevalence of mental illness among LTC recipients.

Various factors, including sex, age, income, comorbidity severity, cancer incidence, and urbanization levels influence LTC service preferences. However, inconsistencies in the findings highlight the need for further research to better understand the complex relationships between these factors and LTC choices.

By expanding the availability of information in this area, healthcare professionals and policymakers can develop tailored strategies to optimize LTC delivery, enhance patient outcomes, and ensure the provision of high-quality and cost effective care to individuals in need. Future studies should strive to address existing knowledge gaps and provide more nuanced insights into the multifaceted nature of LTC decision-making.

## 2. Materials and methods

### 2.1. Research design

This study conducted a secondary data analysis that is a retrospective cohort study. The observation period was from 2007 to 2017; data were obtained from the total population recorded in 2006–2017 in the Health and Welfare Data Science Center.

Propensity score matching with a ratio of 1:1 was employed for those who used LTC services (study group) and those who did not (control group), according to sex, age, income-based premium, CCI, and urbanization level.

### 2.2. Data source and study sample

This study utilizes secondary data analysis, which is a type of retrospective cohort study. This study included patients aged over 50 years from the data of the total population recorded in 2006–2017 in the Health and Welfare Data Science Center. A total of 82,814 participants were observed for at least 4 years. Conversely, patients who were 49 years old and below, who had developed mental illness in 2006, who used LTC services after developing mental illness, or who used such services for less than 1 year during the observation period were excluded.

### 2.3. Variables

Independent variables were categorized into four: (1) Types of LTC services (using inhome care services, using institutional care services, using both inhome and institutional care services, and not using LTC services), (2) Demographic characteristics (sex, age, and premium calculated according to income), (3) Health status [CCI and cancer development (Yes/No)], and (4) Area characteristics (the divisions where participants enrolled in the National Health Insurance (NHI) program, and degree of urbanization). Conversely, dependent variables were categorized into three: (1) Mental illness development (Yes/No), (2) Deceased (Yes/No); and (3) Healthcare costs.

### 2.4. Statistical analysis

Differences between the independent variables (types of LTC services, sex, cancer development, and the divisions where participants enrolled in the NHI program) and dependent variables (mental illness development, deceased, and a higher rate of using medical care) were analyzed using the Chi-square test of independence (*χ*^2^ test). Overly high relevance among independent variables was examined and confirmed by colinearity diagnostics. The relationship of various independent variables with the risk of mental illness development and mortality was assessed using the Cox proportional hazards model, showing the relative mortality risk of using various LTC services. In addition, the GLM was used for multivariate analysis to compare differences between the least squares means of the annual healthcare costs per person in those using LTC services.

## 3. Results

The rate of mental illness development was highest in participants using institutional care services (26.22%) and lowest in those not using LTC services (16.93%). Females (22.77%) had a higher rate than males (16.23%). In terms of age, it was highest in those aged 81 years and above (26.08%) and lowest in those aged 50–60 years (7.99%). Furthermore, those with an income below NT$ 21,009 (inclusive) had the highest rate (19.88%), and those with an income of NT$ 28,801–NT$ 36,300 had the lowest (10.37%). Interestingly, the rate was higher in those without cancer (20.00%) than in those with cancer (10.49%). Those who were enrolled in the NHI program with the Taipei Division had the highest rate (20.98%), whereas those enrolled with the Northern Division had the lowest (16.38%). Participants with a CCI score of 3 points had the highest rate (24.40%), and those with 0–1 point had the lowest (19.20%). In terms of the degree of urbanization, the rate was highest in those residing in highly urbanized towns (20.71%) and lowest in those residing in emerging towns (18.45%).

### 3.1. Synthesized findings

As for the mortality rate, those using both inhome and institutional care services had the highest rate (90.64%), and those not using LTC services had the lowest (46.15%). In terms of sex, males had a higher rate (68.79%) than females. In addition, the mortality rate was highest in those aged 81 years and above (84.46%) and lowest in those aged 50–60 years (38.18%). It was also highest in those with an income of less than NT$ 21,009 (inclusive) (70.15%) and lowest in those with an income of NT$ 28,801–NT$ 36,300 (21.31%). Those with a CCI score of 4 points and above had the highest rate (83.74%), and those with 0–1 point had the lowest (66.98%). The mortality rate was also higher in those with cancer (85.25%) than in those without. Regarding the degree of urbanization, those residing in aging towns had the highest rate (70.90%), and those residing in highly urbanized towns had the lowest (66.52%; Table 1).

**Table 1:** Chi-square test analysis of mental illness development and mortality in matched samples (*N* = 82,814).

Variables	Developed mental illness (*n*)	%	No mental illness (*n*)	%	Value of *p*	Deceased (*n*)	%	Survived (*n*)	%	Value of *p*
Types of long-term care services					<0.0001*					<0.0001*
Not using long-term care services	7,018	16.93%	34,425	83.07%		19,124	46.15%	22,319	53.85%	
Using inhome care services	3,990	19.53%	16,444	80.47%		18,411	90.10%	2,023	9.90%	
Using institutional care services	4,694	26.22%	13,208	73.78%		16,211	90.55%	1,691	9.45%	
Using both inhome and institutional care services	640	21.09%	2,395	78.91%		2,751	90.64%	284	9.36%	
Sex					<0.0001*					0.0011*
Male	6,242	16.23%	32,212	83.77%		26,452	68.79%	12,002	31.21%	
Female	10,100	22.77%	34,260	77.23%		30,045	67.73%	14,315	32.27%	
Age					<0.0001*					<0.0001*
50–60 years	693	7.99%	7,981	92.01%		3,312	38.18%	5,362	61.82%	
61–70 years	1,552	12.94%	10,442	87.06%		6,057	50.50%	5,937	49.50%	
71–80 years	5,578	18.92%	23,898	81.08%		19,535	66.27%	9,941	33.73%	
81 years and older	8,519	26.08%	24,151	73.92%		27,593	84.46%	5,077	15.54%	
Premium calculated according to income					<0.0001*					
Less than NT$ 21,009 (inclusive)	15,765	19.88%	63,521	80.12%		55,661	70.15%	23,683	29.85%	<0.0001*
NT$ 21,010–NT$ 28,800	324	16.80%	1,602	83.20%		475	25.07%	1,418	74.93%	
NT$ 28,801–NT$ 36,300	39	10.37%	339	89.63%		79	21.31%	292	78.69%	
NT$ 36,301–NT$ 45,800	83	15.76%	445	84.24%		119	22.99%	397	77.01%	
More than NT$ 45,801 (inclusive)	131	18.61%	572	81.39%		164	23.74%	526	76.26%	
Charlson Comorbidity Index score					<0.0001*					<0.0001*
0–1 point	13,991	19.20%	58,869	80.80%		48,805	66.98%	24,055	33.02%	
2 points	1,621	23.35%	5,322	76.65%		5,255	75.69%	1,688	24.31%	
3 points	526	24.40%	1,630	75.60%		1,721	79.82%	435	20.18%	
4 points and above	204	23.86%	651	76.14%		716	83.74%	139	16.26%	
Cancer development					<0.0001*					<0.0001*
No	16,096	20.00%	64,372	80.00%		54,497	67.73%	25,971	32.27%	
Yes	246	10.49%	2,100	89.51%		2,000	85.25%	346	14.75%	
Division where participants enrolled in the NHI program					<0.0001*					<0.0001*
Taipei Division	6,285	20.98%	23,667	79.02%		20,422	68.18%	9,530	31.82%	
Northern Division	1,585	16.38%	8,089	83.62%		6,510	67.29%	3,164	32.71%	
Central Division	2,791	19.33%	11,645	80.67%		9,719	67.32%	4,717	32.68%	
Southern Division	2,715	20.33%	10,639	79.67%		9,329	69.86%	4,025	30.14%	
Kaoping Division	2,448	19.24%	10,277	80.76%		8,601	67.59%	4,124	32.41%	
Eastern Division	518	19.38%	2,155	80.62%		1,916	71.68%	757	28.32%	
Degree of urbanization					<0.0001*					<0.0001*
Highly urbanized towns	4,425	20.71%	16,942	79.29%		14,214	66.52%	7,153	33.48%	
Moderately urbanized towns	4,459	19.76%	18,109	80.24%		15,322	67.89%	7,246	32.11%	
Emerging towns	2,383	18.45%	10,535	81.55%		8,762	67.83%	4,156	32.17%	
General towns	2,663	19.23%	11,187	80.77%		9,710	70.11%	4,140	29.89%	
Aging towns	535	19.81%	2,166	80.19%		1,915	70.90%	786	29.10%	
Agricultural towns	1,026	20.40%	4,004	79.60%		3,560	70.78%	1,470	29.22%	
Remote towns	851	19.43%	3,529	80.57%		3,014	68.81%	1,366	31.19%	

*p < 0.0001, indicating statistical significance.

According to the survival analysis using the Cox proportional hazards model (Table 2), the mortality rate was 1.027 times higher in those using institutional care services than in those using inhome care services (the reference group for this category). It was also 1.186 times higher in males than in females. In terms of age, the mortality rate was 5.098 times higher in those aged 81 years and above, 2.962 times higher in those aged 71–80 years, and 1.725 times higher in those aged 61–70 years than in those aged 50–60 years (the reference group). Moreover, the mortality rate was 1.607 times higher in those with a CCI score of 4 points and above, 1.407 times higher in those with 3 points, and 1.277 times higher in those with 2 points than in those with 0–1 point (the reference group). Those with cancer had a mortality rate that was 1.365 times higher than those without cancer. In terms of the degree of urbanization, the mortality rate was 1.494 times higher in those residing in agricultural towns, 1.45 times higher in those residing in aging towns, 1.358 times higher in those residing in remote towns, 1.319 times higher in those residing in general towns, 1.123 times higher in those residing in emerging towns, and 1.034 times higher in those residing in moderately urbanized towns than in those residing in highly urbanized towns (The reference group; Table 2).

**Table 2:** Cox proportional hazards model analysis of mortality in the matched samples (*N* = 82,814).

Variable name	Before calibration			After calibration		
	cHR	95% CI	Value of *p*	aHR	95% CI	Value of *p*
Type of long-term care services						
Not using long-term care services	0.334	0.327–0.342	<0.0001*	0.763	0.492–1.184	**0.2275**
Using inhome care services (ref.)	--	--	**--**	--	--	**--**
Using institutional care services	1.060	1.033–1.087	<0.0001*	1.027	1.001–1.053	0.0421*
Using both inhome and institutional care services	1.038	0.989–1.089	**0.1311**	1.042	0.993–1.093	**0.0976**
Sex						
Male	1.001	0.982–1.020	**0.9054**	1.186	1.163–1.209	<0.0001*
Female (ref.)	--	--	**--**	--	--	**--**
Age						
50–60 years (ref.)	--	--	**--**	--	--	**--**
61–70 years	1.555	1.482–1.631	<0.0001*	1.725	1.644–1.810	<0.0001*
71–80 years	2.592	2.486–2.703	<0.0001*	2.962	2.840–3.089	<0.0001*
81 years and older	4.321	4.146–4.502	<0.0001*	5.098	4.889–5.315	<0.0001*
Premium calculated according to income						
Less than NT$ 21,009 (inclusive)	4.750	4.284–5.267	<0.0001*	5.736	5.171–6.363	<0.0001*
NT$ 21,010–NT$ 28,800 (ref.)	--	--	**--**	--	--	--
NT$ 28,801–NT$ 36,300	0.865	0.660–1.135	**0.2951**	1.097	0.837–1.439	**0.5021**
NT$ 36,301–NT$ 45,800	0.925	0.736–1.163	**0.5066**	1.091	0.867–1.371	**0.4582**
More than NT$ 45,801 (inclusive)	0.932	0.760–1.143	**0.4979**	1.080	0.880–1.324	**0.4614**
Charlson Comorbidity Index score						
0–1 point (ref.)	--	--	**--**	--	--	--
2 points	1.326	1.283–1.370	<0.0001*	1.277	1.235–1.319	<0.0001*
3 points	1.446	1.367–1.531	<0.0001*	1.407	1.329–1.489	<0.0001*
4 points and above	1.681	1.541–1.832	<0.0001*	1.607	1.474–1.753	<0.0001*
Cancer development						
No (ref.)	--	--	**--**	--	--	--
Yes	1.715	1.629–1.805	<0.0001*	1.365	1.296–1.438	<0.0001*
Division where participants enrolled in the NHI program						
Taipei Division (ref.)	--	--	--	--	--	--
Northern Division	1.135	1.099–1.172	<0.0001*	1.129	1.091–1.169	<0.0001*
Central Division	1.165	1.134–1.198	<0.0001*	1.144	1.110–1.179	<0.0001*
Southern Division	1.383	1.345–1.422	<0.0001*	1.168	1.130–1.206	<0.0001*
Kaoping Division	1.121	1.089–1.153	<0.0001*	1.160	1.126–1.196	<0.0001*
Eastern Division	1.206	1.144–1.271	<0.0001*	1.001	0.947–1.059	**0.9624**
Degree of urbanization						
Highly urbanized towns (ref.)	--	--	**--**	--	--	**--**
Moderately urbanized towns	1.057	1.029–1.086	<0.0001*	1.034	1.006–1.063	0.0188*
Emerging towns	1.132	1.098–1.168	<0.0001*	1.123	1.085–1.161	<0.0001*
General towns	1.402	1.361–1.443	<0.0001*	1.319	1.277–1.363	<0.0001*
Aging towns	1.656	1.572–1.745	<0.0001*	1.450	1.370–1.535	<0.0001*
Agricultural towns	1.590	1.527–1.657	<0.0001*	1.494	1.427–1.564	<0.0001*
Remote towns	1.378	1.318–1.440	<0.0001*	1.358	1.297–1.421	<0.0001*

*p < 0.0001, indicating statistical significance.

The mean annual healthcare cost per person was significantly higher in those not using LTC services than in those using inhome care services, institutional care services, and both inhome and institutional care services. It was also significantly higher in males than in females, those aged 71–80 years than in the other age groups, those with cancer than in those without, and those residing in moderately urbanized towns than in those living in other towns (Table 3).

**Table 3:** General linear model analysis of the mean annual healthcare cost per person in the matched samples (*N* = 82,814).

Variable name	Least squares means	Value of *p*
Type of long-term care services		<0.0001*
Not using long-term care services	46,551.0	
Using inhome care services	32,055.3	
Using institutional care services	29,148.6	
Using both inhome and institutional care services	26,757.5	
Sex		<0.0001*
Male	34,308.5	
Female	32,947.7	
Age		<0.0001*
50–60 years	28,993.0	
61–70 years	33,683.8	
71–80 years	36,830.0	
81 years and older	35,005.6	
Premium calculated according to income		**0.1254**
Less than NT$ 21,009 (inclusive)	32,579.0	
NT$ 21,010–NT$ 28,800	33,576.1	
NT$ 28,801–NT$ 36,300	34,565.2	
NT$ 36,301–NT$ 45,800	32,877.8	
More than NT$ 45,801 (inclusive)	34,542.3	
Charlson Comorbidity Index score		<0.0001*
0–1 point	15,760.4	
2 points	30,672.2	
3 points	39,622.1	
4 points and above	48,457.7	
Cancer development		<0.0001*
No	31,426.4	
Yes	35,829.8	
Division where participants enrolled in the NHI program		<0.0001*
Taipei Division	34,002.9	
Northern Division	31,965.9	
Central Division	35,355.2	
Southern Division	32,863.8	
Kaoping Division	33,800.7	
Eastern Division	33,780.0	
Degree of urbanization		<0.0001*
Highly urbanized towns	34,820.8	
Moderately urbanized towns	35,410.8	
Emerging towns	33,624.3	
General towns	33,076.5	
Aging towns	32,161.6	
Agricultural towns	32,935.0	
Remote towns	33,367.6	

*p < 0.0001, indicating statistical significance.

### 3.2. Life expectancy and survival time

Services provided by LTC facilities vary, resulting in differences in mortality risk. As shown in the survival analysis results (Table 2), those using institutional care services had higher mortality risk (1.027 times higher) with a shorter survival time (4.08 years). Compared with those not using LTC services, those using LTC services were more likely to develop mental illness. This study also found that after using LTC services (Table 4), the shortest interval at which an individual developed mental illness was approximately 236 days. Those using both inhome and institutional care services had a survival time of approximately 4.2 years, with a life expectancy rate of approximately 76.9 years, which was only higher when compared with those using institutional care services alone.

**Table 4:** Intervals at which the participants using different types of long-term services develop mental illness, their life expectancy, and their survival time.

Type of long-term care services	Rate of mental illness development	Interval (number of days)	Mortality rate	Age of the participants using long-term care services	Life expectancy	Survival time	Years of life lost
Not using long-term care services	16.93%	282	46.15%	–	78.8	7.2	1.2
Using inhome care services	19.53%	254	90.10%	72.9	77.2	4.3	2.8
Using institutional care services	26.22%	246	90.55%	73.0	77.1	4.1	2.9
Using both inhome and institutional care services	21.09%	236	90.64%	72.7	76.9	4.2	3.1

Life expectancy is calculated according to the average lifespan of 80.4 years in Taiwan announced by the Ministry of the Interior in 2017.

### 3.3. Comparison of benefits between various LTC services

**Figure 1:**
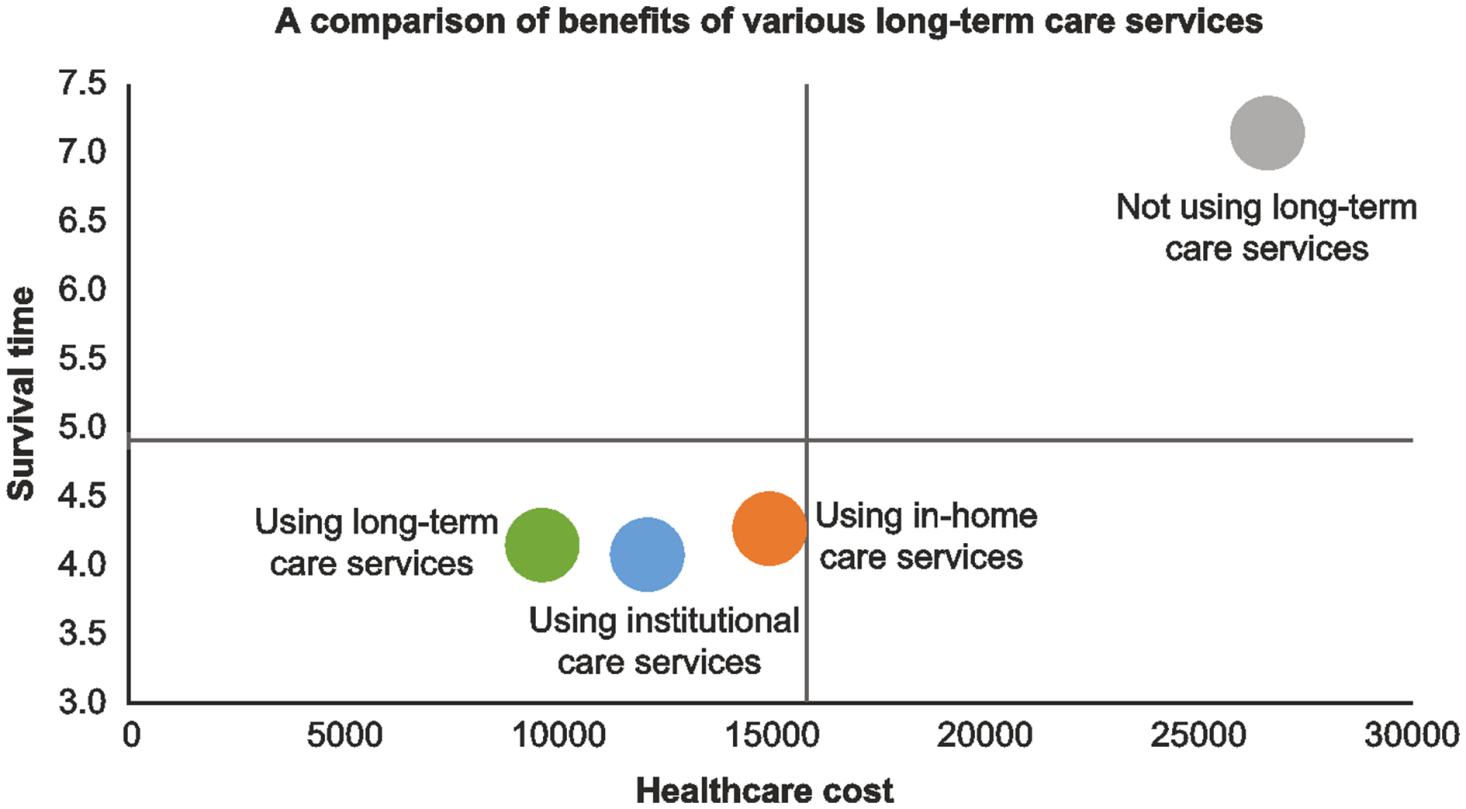
Comparison of benefits between various long-term care services.

This study also provided a comparison graph of the benefits of various LTC services according to the results (Figure 1). Those not using long-term services are in the first quadrant, having the characteristic of bearing higher mean annual healthcare costs with a long survival time. Those using LTC services are in the third quadrant, having the characteristic of bearing low healthcare costs with a short survival time. The graph shows that they are at risk of developing mental illness. However, attention should be paid to those using both inhome and institutional care services because as illustrated, they have a higher risk of developing mental illness and dying from it.

## 4. Discussion

International studies have emphasized that LTC services can prevent diseases or delay deterioration in patients, thereby delaying death. In addition, nursing services are already provided at the initial stage of LTC services (19). When serious changes occur in the activities of daily living for older adults or in their health status, inhome care costs increase significantly (20). Furthermore, the risk of developing dementia in patients with depression is twice than that in those without depression. Dementia decreases the activities of daily living in patients with depression and increases their dependence. It is also closely related to increased mortality rate and increased healthcare costs (21, 22).

The individuals using institutional care services may experience failure in adapting to a new environment Consequently, their mortality rate is higher than that of those using inhome or community-based care services, i.e., individuals who will age in place (23, 24). Given that older adults’ needs for care change along with the course of their diseases, they may switch from inhome to institutional care services. Thus, when switching between different types of LTC services, healthcare providers must consider that mortality risk is potentially associated with this action (25).

This study also revealed that those not using LTC services have the highest mean annual healthcare cost and are also likely to be the most frequent users of medical care services (20.077 times more frequent). Given the lack of professional care resources at home, they need emergency medical care or to be hospitalized when they have health problems. Thus, their healthcare costs are comparatively higher than those of LTC residents (26). Although those using inhome care services receive professional care compared with those not using inhome care services, they usually receive care for only a short period of time, and they will be sent to the hospital for medical care when their caregivers cannot cope with their health problems. Therefore, they bear the second highest healthcare cost. However, when their condition deteriorates to a certain extent, whereby they need emergency medical care, their total medical expenses will increase (27). In contrast, those using both inhome and institutional care services bear the lowest mean annual healthcare cost because of the discontinuity of care.

This study found that females had a higher risk of developing mental illness than males. However, males had higher mortality risk and higher healthcare costs than females. Studies show that females are more likely to develop depression followed by mental illness because of their gender roles, social support, episodes in their life, and mental health status (3, 28).

In addition, those aged 81 years and above had higher risks for mental illness development and mortality; they also bore higher healthcare costs than those aged 71–80 years. International studies have highlighted that mortality risk increases with age (27, 29). In 2006–2017, the life expectancy in Taiwan was approximately 79.34 years. Individuals aged 71–80 years often need medical care and institutional care services because of physical weakness, and LTC costs increase significantly with age (7, 27). When the income-based premium was lower, the risks for mental illness development and mortality increased. International studies suggest that those with a lower income have fewer resources, thereby often deprived to use LTC services. They are also more likely to develop depression when using LTC services. In contrast, those with a higher income have a better chance of delaying death (15, 19, 29). In terms of CCI, those with higher scores have higher risks for mental illness development and mortality while bearing higher healthcare costs. According to international studies, most of those using LTC services will develop dementia, along with chronic diseases, because they generally have higher CCI scores. Their depression severity scores also increase as their CCI scores increase. Given that their condition is more complicated and severe, their survival time will be shortened, and their healthcare costs will also significantly increase (17, 20, 22, 30).

Those without cancer have a higher risk of developing mental illness than those with cancer. However, as expected, those with cancer had higher mortality risk and bore higher healthcare costs than those without cancer. One probable reason on why those without cancer are more at risk of developing mental illness is that they are less knowledgeable of their illness and actively seek medical help in comparison with those with cancer. Considering that cancer is a serious condition, those with this disease have higher mortality risk and higher healthcare costs than those without (7, 31, 32). The present study also showed that those residing in agricultural towns had the highest risks for mental illness development and mortality, and those residing in moderately urbanized towns had the highest healthcare costs. Generally, people residing in areas other than large cities are more likely to be diagnosed with mental illness. In particular, those residing in rural areas with a low pension relatively lack medical resources. Mental health services for older adults are only provided in a few areas, and referral services are often unavailable. Consequently, the treatment needs of those with depression cannot be satisfied; thus, they have higher risks for mental illness development and mortality. Furthermore, areas where people reside affect insurance products that they purchase, as reflected in the differences noted in the types of LTC services availed (9, 15, 17, 22, 33).

This study focuses on prioritizing interventions for LTC recipients with high mortality risk but low healthcare utilization, particularly those who reside in institutional settings and often have severe functional impairments. Aside from addressing the physical health needs of older individuals, paying close attention to their mental well-being is recommended. Beyond providing basic care, healthcare providers should offer emotional support and encourage family members to spend time with their loved ones in the institutional setting, aiming to reduce older patients’ feelings of anxiety and abandonment and minimize depression occurrence.

This article discusses two limitations and potential variations in the generalizability of the study findings. First, the study utilized data from the Health Insurance System provided by the Ministry of Health and Welfare; this system only includes information on patients who have utilized healthcare resources. Consequently, data related to individuals who privately fund their LTC or hire private caregivers were not included in the dataset. Second, the study only focused on the context of Taiwan; the possibility of having some differences in other countries should be acknowledged.

This study presents recommendations for future research and analysis in LTC and mental health. First, establishing a link between the Health Insurance Database and other LTC databases is recommended to gather more comprehensive data. This linkage will provide a more holistic understanding of the factors influencing mental health outcomes in individuals receiving LTC services. Additionally, future studies may conduct detailed analyzes of specific mental illnesses such as anxiety disorders, schizophrenia, depression, bipolar disorder, substance abuse, and dementia. Information on mental health conditions with a greater impact can serve as a valuable reference for healthcare authorities and medical institutions.

In conclusion, older adults using inhome and institutional care services might have a greater risk of developing mental illness with a shorter survival time. Those residing in agricultural towns are the most at risk for mental illness development and mortality, whereas those residing in moderately urbanized towns bear higher healthcare costs and have a higher rate of utilizing medical care. These results may serve as reference for units in charge of health policies and be used by LTC providers as the direction of intervention and reference indicator when formulating policies.

## Data availability statement

The raw data supporting the conclusions of this article will be made available by the authors, without undue reservation.

## Ethics statement

The studies involving humans were approved by the current study was reviewed and approved by the Research Ethics Committee at China Medical University and Hospital, Taichung, Taiwan. The studies were conducted in accordance with the local legislation and institutional requirements. The participants provided their written informed consent to participate in this study.

## Author contributions

C-LW and J-YW conceptualized and designed the study. C-LW performed the data analysis. P-YT and C-LW interpreted the data and drafted the manuscript. P-YT and J-YW evaluated and revised the manuscript. J-DC, K-JM, C-YY, and J-YW coordinated and supervised the study and provided critical feedback. All authors contributed to the article and approved the submitted version.
